# Population Ecology of Glacier Bacteria from the View of Gene Flow in *Cryobacterium*

**DOI:** 10.3390/microorganisms14020326

**Published:** 2026-01-30

**Authors:** Jiayu Hu, Yujie Du, Jihua Hu, Luyao Zhang, Yongjie Wu, Yilin Shu, Liang Shen

**Affiliations:** Anhui Provincial Key Laboratory of Molecular Enzymology and Mechanism of Major Metabolic Diseases, College of Life Sciences, Anhui Normal University, Wuhu 241000, China

**Keywords:** *Cryobacterium*, extreme cold ecosystems, ecological unit, gene flow, glacier habitat, selective sweeps

## Abstract

Glaciers have been proposed as evolutionary hotspots for microbial evolution; however, direct evidence for glacial microbial population formation and genomic loci undergoing selective sweeps remains limited. To address this knowledge gap, we investigated the genomic diversity, evolutionary pressures, and adaptive strategies of *Cryobacterium*, a representative genus of glacier environments. Based on recent gene flow analysis, 18 distinct populations of *Cryobacterium* were identified, each exhibiting clear discontinuities in gene flow and genetic boundaries. Selective pressure analyses revealed purifying selection within populations, maintaining genetic stability, and positive selection between populations, suggesting adaptive divergence from environmental differences. Notably, half of the populations spanned geographically distant glaciers, suggesting widespread dispersal mechanisms such as atmospheric circulation or glacial fauna migrations. We identified 17 genes under strong selective sweeps, involved in metabolic enzymes, transporters, and gene regulation. Based on the reverse ecology principles, these genes (e.g., glucose-6-phosphate dehydrogenase assembly and RNA polymerase-binding gene), are likely to be critical for cold adaptation. This study provided clear genomic evidence of glacial microbial population formation driven by recent gene flow, significantly enhanced our understanding of microbial adaptation in extreme cold ecosystems, and emphasized the importance of deep genomic sequencing in ecological and evolutionary research.

## 1. Introduction

Ecological processes reflect the dynamic interactions between organisms and their environment, with populations serving as the fundamental units for understanding adaptation and divergence [1]. Defining microbial populations, however, remains challenging due to their genetic plasticity, rapid evolution, and horizontal gene transfer [2,3]. Despite the powerful framework that population genomics provides for delineating ecological units, its application remains limited in extreme environments particularly glacial ecosystems [4].

Recent progress in reverse ecology has enabled the identification of microbial populations by analyzing patterns of recent gene flow. This approach relies on the principle that genomic regions transferred horizontally exhibit lower levels of SNPs (Single Nucleotide Polymorphisms) accumulation than vertically inherited sequences [1,5]. Tools such as PopCOGenT implement this methodology and have been validated in model systems including *Vibrio*, *Sulfolobus*, and *Prochlorococcus* [6,7,8], as well as in clinically relevant settings, such as in populations of *Ruminococcus gnavus* associated with inflammatory bowel disease [9]. We wonder whether there are scenarios that could lead to identical genomic regions between closely related organisms from geographically disparate locations [10]. For instance, distantly separated species subjected to similar environmental pressures, such as low temperature in glacier environments, might share identical genomic fragments and conserved genomic features [11].

Glaciers constitute a dynamic, “extremely fluctuating” cold environment characterized by persistently low temperatures, frequent freeze–thaw cycles, high solar radiation, oligotrophic conditions, and episodic meltwater inputs [12,13]. Importantly, these environmental stressors are not uniform across the global cryosphere [14]. They vary substantially between polar regions (e.g., Antarctica and the Arctic) and high alpine glaciers distributed across major mountain systems such as the Tibetan Plateau and Xinjiang [15]. Such spatial heterogeneity creates a broad spectrum of selective pressures that are likely to drive microbial divergence [16]. How the distinctive, selective regime imposed by glaciers and their microbial inhabitants acts upon key genes and shapes community structure remains largely unexplored [17]. A major reason for this knowledge gap is the rarity of microbial taxa exhibiting glacier-specific phenotypic adaptations. This limitation has hindered the identification of genes and mechanisms underpinning microbial survival and evolution in glacial ecosystems [18]. Consequently, understanding the genomic and functional basis of microbial adaptation to these extreme conditions requires further exploration through advanced genomic and ecological studies.

Across environmentally heterogeneous glacial systems, the spatial distribution of gene flow between distant regions remains to be fully characterized; and it is also unclear whether this flow delineates distinct population boundaries while simultaneously establishing a network of genetic connectivity across remote glaciers. Furthermore, among genomic segments frequently transferred horizontally, the identities of widely disseminated genes that carry key adaptive functions represent a critical area for systematic investigation [19].

Population genomic analysis was performed on 95 high-quality, de-replicated *Cryobacterium* genomes drawn from GenBank and prior cultivation studies (complete metadata and references are provided in Table S1). The PopCOGenT algorithm resolved these genomes into 18 discrete populations [1]. *Cryobacterium* (phylum *Actinomycetota;* class *Actinomycetes;* order *Micrococcales;* family *Microbacteriaceae*) strains are typically isolated from cold environments in polar and high alpine regions (the Arctic, Antarctica, and Tibetan Plateau) [20]. The cold polar and alpine regions function as isolated habitats, analogous to islands, with *Cryobacterium* species representing indigenous microbial communities that are uniquely adapted to these cold environments. Our study provides a good and unique model for investigating the formation of bacterial populations and their ecological roles.

## 2. Material and Methods

### 2.1. Preparation of Cryobacterium Genomes for Analysis

Our dataset is mainly composed of two parts. One part was constructed by supplementing the previously sequenced *Cryobacterium* genomes. As part of our ongoing research into glacial microbiomes, we isolated and sequenced multiple *Cryobacterium* strains from ice and sediment samples. These samples were collected from major cryospheric regions, including high alpine glaciers on the Tibetan Plateau (Xinjiang No. 1 glacier, Touming Mengke glacier) and polar regions. Detailed protocols for sample collection, strain isolation, and genome sequencing are described in Shen et al. (2020) [21]. Briefly, ice core or sediment samples were aseptically collected from glacier surfaces, serially diluted, and plated on low-nutrient media at 4 °C. Colonies were purified and identified via 16S rRNA gene sequencing. Genomic DNA was extracted using the TIANamp Bacteria DNA Kit (Tiangen, Beijing, China) following the manufacturer’s instructions, and sequenced on the Illumina Hiseq 2000 platform.

**Figure 1:**
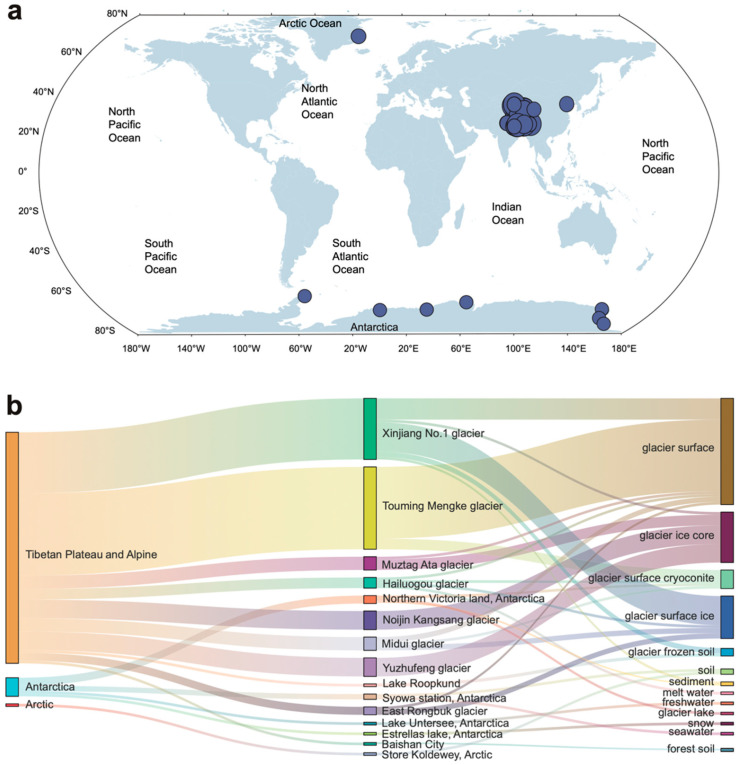
**Geographical location and isolation source of the** ***Cryobacterium*** **isolates analyzed in this study.** (**a**) The *Cryobacterium* isolates were mainly from the Arctic, Antarctic, and Tibetan Plateau, except *Cryobacterium soli* GCJ02 from the forest soil of Baishan, Jilin, China. (**b**) Sankey diagram showing the ecosystem, ecosystem subtype, and specific sample types of the *Cryobacterium* isolates.

The other part contains publicly available genomes. We retrieved all genomes with the taxonomic identifier ‘*Cryobacterium*’ from the NCBI GenBank database (accessed in November 2023). This yielded an initial set of 161 genomes from worldwide glacial environments (Table S1, Figure 1a).

Due to the incomplete classification of this group, the taxonomic information of the original genomes was reclassified using GTDB-Tk [22]. Genome completeness and contamination were calculated using CheckM v1.0.7 (under default setting) [23]. Genome quality filtering and dereplication were performed according to Parks et al. (2017) [24] and Shen et al. (2021) [25]. Genomes with a length exceeding 300 contigs, an N50 smaller than 20 kb, a completeness lower than 95%, and a contamination rate higher than 5% were removed. The genomes were de-replicated at an average amino acid identity (AAI) cutoff of ≥99.5%. The AAI value was calculated using CompareM (default options) (https://github.com/dparks1134/CompareM, accessed on 15 October 2023). In the end, 95 genomes were obtained. Detailed information on the data source, biogeographic distribution, and genomic quality can be found in Table S1. Detailed information on minimum average amino acid identity (AAI) and minimum Average Nucleotide Identity (ANI) in each population can be found in Table S2.

### 2.2. Phylogenetic and Genomic Analyses

Outgroup species that are closely related to the ingroup species are more suitable for phylogenetic reconstruction than distantly related species or ingroup species [26]. Thus, for phylogenomic clustering, complete genomes of *Clavibacter michiganensis* CFBP7494 (GCF_002151165.1) and *Clavibacter michiganensis* Z001 (GCF_002931335.1) were chosen as the outgroup species as they are close relatives of *Cryobacterium* in the family *Microbacteriaceae* [27]. With the two *Clavibacter* and 95 *Cryobacterium* genomes, a maximum-likelihood phylogenomic tree was constructed using PhyloPhlAn3 with default options [28]. As the phylogenomic tree can be drawn in multiple different equivalent appearances, the tree was sorted with increasing node order using FigTree 1.4.4 to obtain a relatively fixed topology (https://github.com/rambaut/figtree/releases, accessed on 15 October 2023).

### 2.3. Recent Gene Flow Detection with PopCOGenT

The identification of recent gene flow events was performed using PopCOGenT (https://github.com/philarevalo/PopCOGenT, accessed on 15 October 2023) [1]. The PopCOGenT program detects the existence and intensity of gene flow by analyzing the length distribution of identical sequence fragments between pairs of genomes and was employed to estimate recent horizontal gene flow among co-existing microbial genomes. The pipeline proceeds in three main steps: (1) Detection of very recent horizontal gene transfer (HGT). For every pair of genomes, we screen the core alignment for unusually long, 100% identical segments that are significantly longer than the background tract length expected from neutral mutation (PopCOGenT default: ≥500 bp with *p* < 0.01 under a Bernoulli null). These segments are taken as evidence of recent DNA exchange that occurred after the two lineages began to diverge. (2) Construction of a recent gene flow network. Each genome is a node; an edge is drawn if the two genomes share no less than the defined length threshold of recently transferred DNA. Edge weights are proportional to the total length of these shared tracts. (3) Identification of gene flow discontinuities. The network is partitioned with the Infomap algorithm [29]. Modules that are internally well connected but separated from other modules by gaps in recent gene flow are defined as PopCOGenT populations. Hence, the absence (not the presence) of ongoing recombination delimits populations, thereby turning gene flow from a confounding factor into the decisive signal. We used this tool to cluster and analyze all genomes, operating with default parameters, as detailed in the Supplementary Materials (File S1).

Further analysis of significant matches was conducted using an aligned tree constructed from multiple sequence alignments to detect phylogenetic inconsistencies, thereby identifying potential transfer elements or indicating potential integration sites for HGT. PopCOGenT v1.0 relies on several external tools, including Mugsy (https://mugsy.sourceforge.net/, accessed on 15 October 2023), a multiple whole-genome alignment tool; Infomap (https://www.mapequation.org/infomap/, accessed on 15 October 2023), a network clustering tool; and Muscle (https://www.drive5.com/muscle/, accessed on 15 October 2023), a multiple sequence alignment tool. These tools were separately configured and installed to ensure the normal operation of PopCOGenT. Manuals for using PopCOGenT and custom scripts are available in our repository at https://github.com/environmental-genomes/Cryobacterium, accessed on 15 October 2023. For detailed information on the core gene sweep in each *Cryobacterium* genome, please refer to Table S3.

### 2.4. Genomic Comparison and Diversity Analyses

KaKs_Calculator v3.0 (https://ngdc.cncb.ac.cn/biocode/tools/BT000001, accessed on 15 October 2023) was used to calculate Ka and Ks for the coding sequence (CDS) files generated after aligning the genomic data. ANI was calculated using the ANI calculator (http://enve-omics.ce.gatech.edu/ani/, accessed on 15 October 2023). AAI values were calculated using CompareM with default options (https://github.com/dparks1134/CompareM, accessed on 15 October 2023). To explore the nucleotide diversity, we used PopCOGenT to calculate the nucleotide polymorphism and genetic diversity. We performed analyses at both the population level and within specific genomic regions identified by the alignment algorithm. Due to the influence of genetic recombination, mutations, or other evolutionary events, these regions may vary across genomes. The identification process involves aligning multiple genomic sequences to determine the evolutionarily conserved regions and the regions with differences among individuals.

## 3. Results

### 3.1. Overview of the Cryobacterium Genomes

After quality filtering and de-replication (see Methods for detail), a total of 95 non-redundant genomes (including 12 genomes of type strains) were obtained, mainly originating from the Tibetan Plateau, Arctic, and Antarctic regions. The majority of genomes (*n* = 87, 92%) were derived from high alpine glaciers on the Tibetan Plateau, with Antarctica contributing 7% (*n* = 7) and the Arctic just 1% (*n* = 1).

Almost all *Cryobacterium* strains are isolated from the cold polar and alpine regions (Figure 1a), except for the strain *Cryobacterium soli* GCJ02, which was isolated from forest soil in Baishan, Jilin, China. The dataset is primarily composed of strains from the Xinjiang No. 1 glacier and Touming Mengke glacier (Figure 1b).

The size of *Cryobacterium* genomes ranged from 3.20 Mb (*Cryobacterium* sp. TMT2-48-2, isolated from an ice sample from the Touming Mengke glacier) to 4.71 Mb (*C. melibiosiphilum* Hh39, isolated from an ice sample from the Xinjiang No. 1 glacier), and averaged at 3.80 Mb in size. The GC contents varied from 61% (*Cryobacterium* sp. Y29, isolated from an ice core of the Yuzhufeng glacier) to 69% (*Cryobacterium* sp. Hh4 and *Cryobacterium* sp. RHLT2-21, isolated from the Xinjiang No. 1 glacier and Hailuogou glacier), averaging 65% (Table S1).

### 3.2. Identification of Cryobacterium Populations

**Figure 2:**
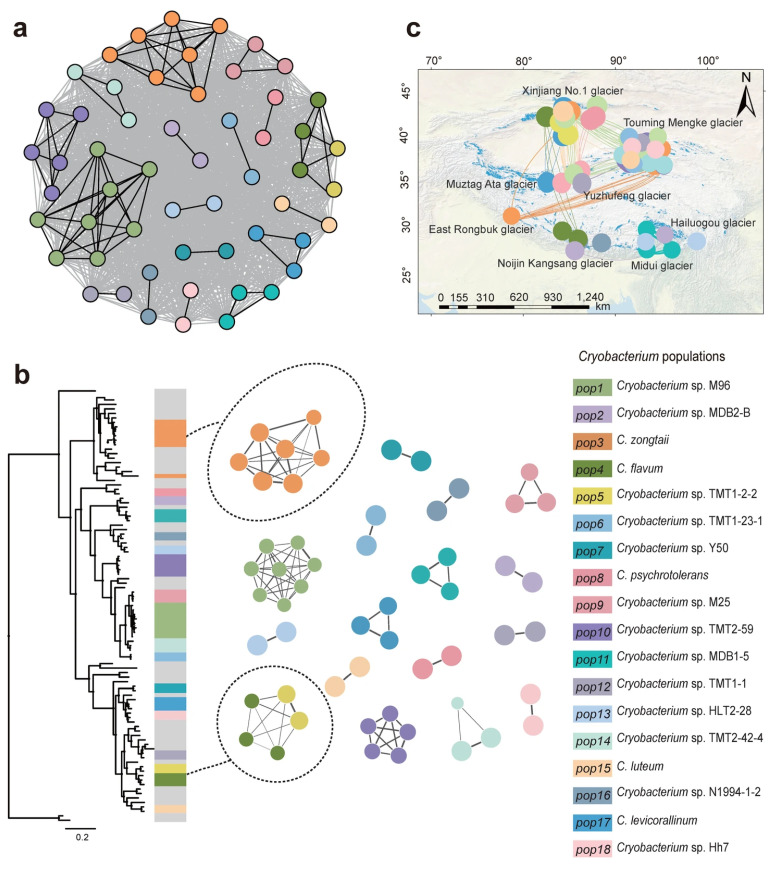
**Clusters of** ***Cryobacterium*** **populations in networks of recent gene flow.** (**a**) Genetic interaction network of the *Cryobacterium* isolates with identifiable recent gene flow. The color of the strains is assigned according to the clustering of PopCOGenT. The edges represent the closest gene flow between genomes. Gene flows with a length bias greater than 10 are represented by black edges, and gray edges represent those with a length bias less than 10. According to the PopCOGenT definition of populations, gene flows with a length bias greater than 10 occur within populations, while gene flows between populations are characterized by a length bias of less than 10. (**b**) Phylogenetic tree of the evolutionary relationship of *Cryobacterium* species, integrated with the gene flow networks of 18 population clusters (pop1–pop18). Using the Infomap method, different strains perform random walks in the network, with the shortest encoding used to describe the path and identify independent population clusters. The size of the nodes corresponds to the genome flow of the strains, which were randomly selected as representatives of each population with the priority given to type strains. (**c**) Mapping *Cryobacterium* populations to the geographical location of each isolate and the source glaciers. The connections between the circles represent recent gene flow as described.

PopCOGenT analysis identified recent gene flow signals within a subset of 55 out of the 95 *Cryobacterium* genomes. These 55 genomes formed a fully connected gene flow network at the broadest scale, yet the analysis resolved 18 distinct populations (pop1–pop18) based on a pronounced length bias threshold (Figure 2a). Gene flow events within each population consistently exhibited a length bias of >10, indicative of strong intra-population genome homogenization, whereas inter-population gene flow events showed a length bias of <10, confirming their relative genetic independence. Recent gene flow exhibited a clustering pattern that is highly consistent with the phylogenetic cluster profile. Except for one strain in the population pop3, 17 populations, out of the 18, formed monophyletic clades.

Notably, significant gene flow was observed between two distinct populations, pop4 and pop5. These populations consist of strains originating from different glaciers; pop4 includes genomes from the Noijin Kangsang and Xinjiang No. 1 glaciers, while pop5 represents strains from the Touming Mengke glacier (Figure 2b).

The 18 populations displayed contrasting geographical distribution patterns (Figure 2b). Nine populations (pop5, 6, 7, 8, 10, 11, 13, 14, and pop16) were glacier-specific, comprising strains all isolated from the same glacier, while the remaining nine populations (pop1, 2, 3, 4, 9, 12, 15, 17, and pop18) contain strains sourced from multiple glaciers (Figure 2c).

### 3.3. Genomic Variation and Evolutionary Selection Patterns of Cryobacterium Populations

**Figure 3:**
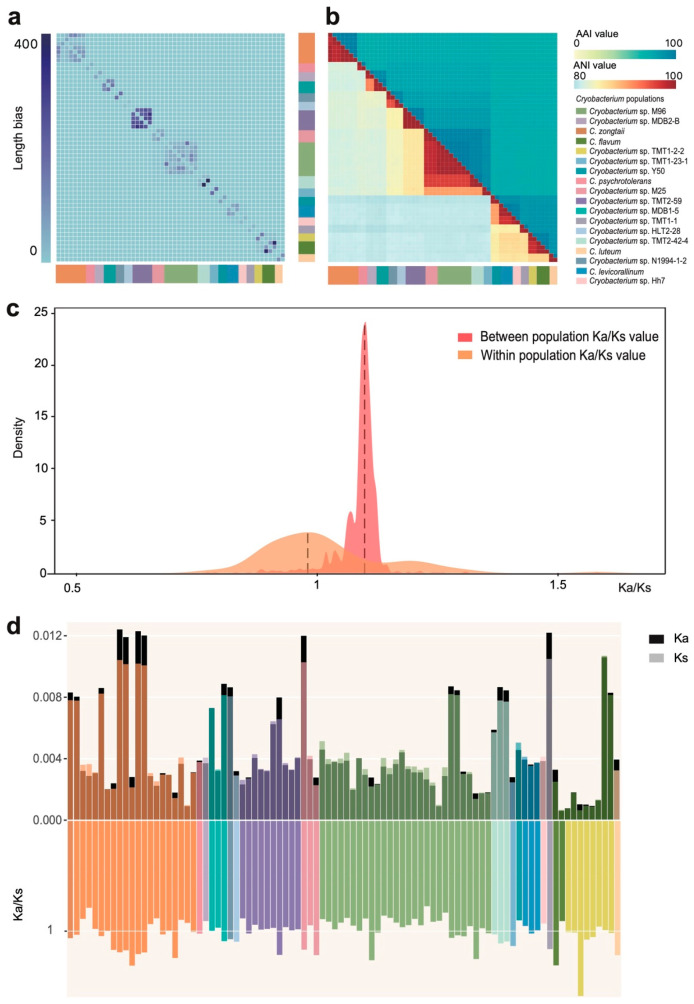
**Assessment of genetic identity and evolutionary trajectory of the genome of** ***Cryobacterium*** **based on gene flow and selection pressure.** (**a**) Heat map of paired genome alignment length bias of the *Cryobacterium* strains based on cluster analysis. (**b**) Heat map constructed with Average Nucleotide Identity (ANI) and average amino acid identity (AAI); the lower triangle of the heat map shows the ANI value, while the upper triangle shows the AAI value. The arrangement of the 55 genomes was based on the phylogenetic order in Figure 1b. (**c**) Distribution of the Ka/Ks ratio between (red) and within (brown) *Cryobacterium* populations. The dotted lines indicate the peak location of the distribution. (**d**) Bar chart illustrating the non-synonymous substitution rates (Ka), synonymous substitution rates (Ks), and their ratios (Ka/Ks) of the 55 *Cryobacterium* isolates. The stacked bar chart above shows the distribution of Ka (dark color) and Ks (light color) values in 55 genomes of *Cryobacterium* species sequenced according to the evolutionary tree in 1b. The correlation between Ka, Ks, and Ka/Ks ratios was assessed based on genome sequencing. The bar chart below shows the overall distribution of Ka/Ks ratios.

The length bias of genomic regions among the populations ranged from 13.1 to 486.2 (Table S2), showing a similar clustering profile to the population structure (Figure 3a). Both ANI and AAI values showed clear boundaries between populations, and high identity within populations (Figure 3b). The minimum ANI and AAI within populations was higher than 97.82% and 97.54% (Table S4). The overall distribution of the Ka/Ks ratio within populations was slightly lower than one, while between populations it was higher than one (Figure 3c).

To quantify selective pressures within populations, we performed a pairwise Ka/Ks analysis for all genomes belonging to the same population. Specifically, for each of the 18 populations, we calculated the average Ka/Ks ratio between every possible pair of its member genomes. The sum of all such intra-population comparisons across the entire dataset amounted to 90 unique genome pairs. At the individual population level, the Ka values ranged from 0.0006 in the *Cryobacterium* sp. TMT2-59 population to 0.0124 in the *C. flavum* population. The *Cryobacterium* sp. TMT2-59 population exhibited the lowest Ks value (0.0007), while the *C. psychrotolerans* population had the highest Ks value (0.0107). Among these 90 gene pairs, 43 had a Ka/Ks ratio greater than 1, with the highest value being 1.58, while 47 had a Ka/Ks ratio less than 1, with the lowest value being 0.78 (Figure 3d).

### 3.4. Genetic Diversity and Core Gene Sweeps of Cryobacterium Populations

**Figure 4:**
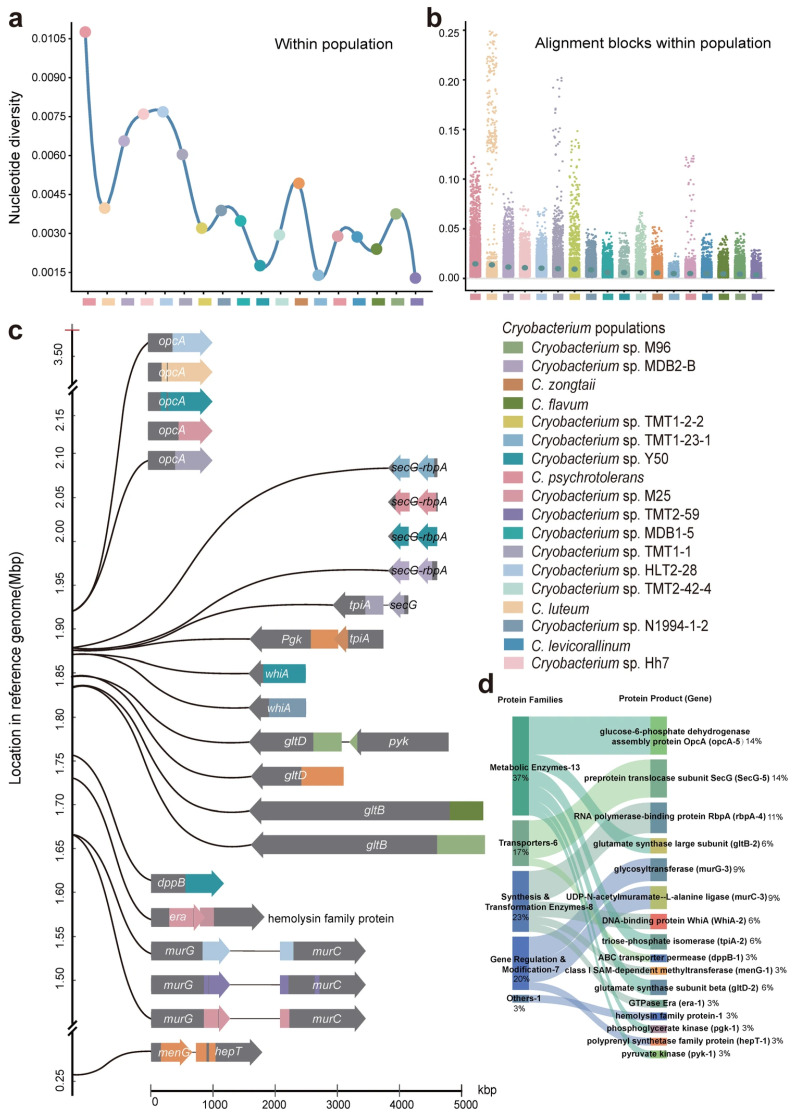
**Nucleotide diversity within populations and alignment blocks of the** ***Cryobacterium*** **and loci that have recently undergone population-specific selective sweeps are restricted to specific genes and functional categories.** (**a**) The overall genomic diversity of the population. Populations are ranked along the x-axis in descending order of their average nucleotide diversity (π) calculated from aligned genomic blocks. The line chart reveals the variation trend of nucleotide diversity among different cryogenic bacterial populations by connecting each population with a smooth curve. The color coding is consistent with the left panel to differentiate between different populations. (**b**) The nucleotide diversity of various alignment blocks within each population. Each point indicates the degree of nucleotide genetic diversity of a comparison block in a specific population. The blue–green dots in the band represent the average of all the matching block nucleotides. (**c**) Location of the sweep regions shown in the reference genome of *Cryobacterium* sp. LW097; loci that have undergone recently population-specific selective sweeps are restricted to specific genes, sweeps from population 1 to 18 are shown as indicated by color key in the middle left. (**d**) Distribution of the genes that have undergone selective sweeps in functional categories. The Sankey diagram illustrates the functional classification of the 17 core genes exhibiting selective sweeps. The width of each flow corresponds to the number of genes, with exact counts and percentages labeled for both functional categories (left) and individual genes (right). Color coding represents different types of genes.

The population of *C. psychrotolerans* from the Xinjiang No. 1 glacier contain two genomes (*C. psychrotolerans* and *Cryobacterium* sp. Sr8), which exhibited the highest nucleotide divergence (*π* = 0.011), despite originating from the same geographic area. In contrast, the *Cryobacterium* sp. TMT2-59 population from the Touming Mengke glacier, comprising five genomes (*Cryobacterium* spp. TMT2-10, TMT4-10, TMT2-23, TMT1-21, and TMT2-59), demonstrated the lowest intra-population nucleotide diversity (*π* = 0.001) (Figure 4a).

Further analysis of nucleotide diversity in comparison blocks revealed distinct patterns among populations. The *C. psychrotolerans* population (Xinjiang No. 1 glacier) contained a large number of alignment blocks with diversity values exceeding 0.011, with most diversity values concentrated close to this value. In contrast, the *C. luteum* population (Xinjiang No. 1 glacier and Touming Mengke glacier) had lower nucleotide diversity among comparison blocks but exhibited higher variation, with a diversity gap between 0.025 and 0.125. In contrast to these highly diverged populations, the *Cryobacterium* sp. M96 population (Touming Mengke glacier, Muztag Ata glacier, Xinjiang No. 1 glacier) and *Cryobacterium* sp. TMT2-59 population (Touming Mengke glacier) showed a much lower level of nucleotide diversity, with the majority of comparison blocks having diversity values less than 0.001 (Figure 4b).

Seventeen genes (including *secG*, *rbpA*, *tpiA*, *pgk*, *whiA*, *gltB*, *gltD*, *dppB*, *era*, *murG*, *murC*, *menG*, *hepT*, *opcA*, *ftsW*, *pyk*, and a hemolysin family protein gene) were identified as having undergone strong selective sweeps, with *opcA*, *secG*, and *rbpA* being the most frequently swept (Table S4). These genes are implicated in fundamental processes for survival in glaciers, central metabolism and stress response (*opcA*, *pgk*, *pyk*), protein synthesis and folding (*secG*, *rbpA*), cell wall/membrane integrity (*murC*, *murG*, *hepT*), nutrient transport (*dppB*, *gltB/D*), and gene regulation (*whiA*). Their prevalence suggests a concerted genomic adaptation to combined stresses of cold, oligotrophy, and high radiation. These genes may play the most critical role in the adaptation of these populations to the cold–oligotrophic glacial environment (Figure 4c).

Genes in the 55 genomes that were predicted to have undergone selective sweeps, and their products, were classified into five main functional categories: ‘metabolic enzymes’ (13 genes), ‘transporters’ (6 genes), ‘synthesis and transformation enzymes’ (8 genes), ‘gene regulation and modification’ (8 genes), and ‘hemolysin family proteins’ (1 gene). ‘Metabolic enzymes’ were the most abundant functional category, particularly glucose-6-phosphate dehydrogenase assembly protein OpcA, preprotein translocase subunit SecG, and RNA polymerase-binding protein RbpA. Key enzymes in amino acid metabolism, such as glutamate synthase, which plays a central role in nitrogen metabolism and amino acid synthesis, were also prominent (Figure 4d).

## 4. Discussion

### 4.1. Defining the Cryobacterium Populations

In this study, we detected recent gene flow in 55 of 95 *Cryobacterium* strains. Gene flow discontinuities delineated 18 populations from the 55 strains. None of the populations harbor more than one type of strain, suggesting that the populations defined by recent gene flow correspond well to species. Nearly all of the populations (17/18) are monophyletic clusters. Decay in genome alignment length bias and sequence identity showed clear boundaries between the identified populations (Figure 3a,b). This provides strong genetic evidence that bacteria species, and by extension populations, could be well defined, and have significant barriers to gene flow like reproductive isolation in sexual organisms [30]. Indeed, purely asexual bacterial species are rare in nature.

The minimum genome sequence identity within the *Cryobacterium* populations is 97.82% (ranging from 97.82% to 99.45%), higher than the conventional 95% genome sequence identity threshold for defining operational bacterial species [31]. Our result is consistent with the view that the commonly applied taxonomic threshold of approximately 95% ANI for delineating bacterial species fails to adequately reflect the degree of genetic divergence that establishes a significant barrier to gene flow between species [32]. In ecological practice, defining species based on gene flow provides higher accuracy and flexibility compared to the use of fixed thresholds. This underscores the importance of the deep sequencing of environmental samples to obtain sufficient whole genomes or metagenome-assembled genomes (MAGs), and highlights the importance of sustained investment in this endeavor.

### 4.2. Ecology of Cryobacterium Populations

The scientific community has long proposed that glacial ecosystems are hotspots for life evolution and species formation [17]; however, few studies have directly tested this hypothesis. We identified very recent gene flow from the glacier-indigenous *Cryobacterium*, which are common in cold environments but rarely detected in temperate or tropical regions [33]. The recent gene flow in *Cryobacterium* indicates that it is evolutionarily active. This is further supported by the selective pressure analysis. The analysis of Ka/Ks ratios revealed distinct evolutionary pressures within and between populations. The overall distribution of Ka/Ks ratios within populations was slightly lower than one, indicating the presence of purifying selection. This suggests that deleterious nonsynonymous mutations are being selectively removed to maintain gene function and genetic stability within populations [34]. In contrast, the Ka/Ks ratios between populations were higher than one, indicative of positive selection, which implies that nonsynonymous mutations conferring adaptive advantages are being retained, potentially driving continuous population differentiation and adaptation to diverse glacier habitats [34].

We found that half of the populations spanned geographically distant glaciers (e.g., between the East Rongbuk glacier in the southwest of the Tibetan Plateau and the Touming Mengke glacier in the northeast), a scenario that had not been accounted for in the study conducted by Arevalo et al. (2019) [1]. This finding highlights the potential for widespread dispersal and connectivity among glacial microbial populations. The exact mechanism of gene flow in the absence of physical contact needs further investigation [1,9]. Several hypotheses could explain this phenomenon: Species originating from the same glacier may disperse via atmospheric circulation or the migration of glacial fauna [35]; these species may have independently evolved but retained identical genomic regions due to convergent environmental pressures, which are then identified as sweep regions by the PopCOGenT algorithm. The first hypothesis is supported by the fact that the populations with the highest nucleotide diversity all originated from the Xinjiang No. 1 glacier. The trend of classifying bacterial taxonomy into monophyletic groups stems from the concept that species originate from a single common ancestor. If this holds true, *Cryobacterium* represents a monophyletic lineage derived from a single evolutionary origin, followed by global dispersal and adaptation to diverse, low-temperature habitats.

### 4.3. Genome Loci That Have Undergone Selective Sweeps in the Cryobacterium Populations

Gene flow was interpreted across the entire genome without prior notion of what is a core or flexible genome, thus gene flow can be used to both define populations and assess the adaptive potential of the entire set of genes [1]. In the 18 *Cryobacterium* populations, population-specific selective sweeps are restricted to specific genes. The products of these genes mainly belonged to four functional categories, including metabolic enzymes, transporters, synthesis and transformation enzymes, as well as gene regulation and modification.

The most abundant functional category was ‘metabolic enzymes’, particularly glucose-6-phosphate dehydrogenase assembly protein OpcA [36], which provides essential reducing power (NADPH) in glucose metabolism and supports cellular energy balance. This protein not only facilitates normal cellular metabolism but also helps in maintaining energy balance within cells. Another key protein in this category is preprotein translocase subunit SecG, which is part of the Sec transportase complex and is responsible for transporting synthetic precursor proteins from the cytoplasm to the membrane or external environment [37]. SecG works in concert with other subunits, such as SecE and SecY, to form transmembrane channels, ensuring efficient protein transport across the cell membrane.

RNA polymerase-binding protein (RbpA) is the most abundant protein within the synthesis and transformation enzymes functional category. RbpA plays a key role in acid tolerance and transcriptional regulation by binding to RNA polymerase, enhancing its affinity to specific DNA templates, and facilitating the initiation of transcription [38].

The functional category ‘amino acid metabolism’ is also crucial for nitrogen metabolism [39], with key enzymes such as glutamate synthase playing an important role [40]. These enzymes regulate the synthesis and metabolism of glutamate, ensuring that bacteria can effectively use nitrogen sources and maintain normal growth and metabolism.

In addition to these proteins, the core genome of *Cryobacterium* contains several other proteins related to metabolism, cell structure, material transport, gene regulation, and adaptive responses. For example, enzymes involved in energy metabolism (such as phosphoglycerate kinase, pyruvate kinase, triose-phosphate isomerase), cell wall proteins associated with lipid synthesis (e.g., the polyprenyl synthetase family protein, UDP-N-acetylmuramate: L-alanine ligase, and glycosyltransferase), and material transporter permease (such as ABC transporter permease) all contribute to the survival and adaptation of psychrophilic bacteria in cold environments. Proteins involved in gene regulation and epigenetic regulation (DNA-binding protein WhiA, class I SAM-dependent methyltransferase) also show important roles at low temperatures [41]. Proteins associated with the cell cycle and protein synthesis, such as GTPase Era, are essential for the survival and adaptive strategies of psychrophilic organisms [42].

The distribution of these proteins is closely related to the physiological adaptability of psychrophilic bacteria in cold environments. They contribute to the survival of psychrophilic bacteria in low-temperature environments by optimizing energy metabolism, maintaining cell membrane stability, enhancing material exchange efficiency, regulating gene expression, and promoting stress response. Specifically, metabolism-related enzymes help bacteria maintain efficient energy production, cell wall synthesis proteins ensure the integrity of cell membranes and walls, and transporters enhance the efficiency of material exchange. Gene regulatory proteins respond to low-temperature pressure by regulating transcription activities, which enhances the adaptability of *Cryobacterium* to cold environments. Overall, breaking through energy barriers and maintaining protein transcription and translation at low temperatures are the core challenges that psychrophilic bacteria must address.

## 5. Conclusions

In summary, our study provides genetic evidence supporting species delineation based on gene flow, highlighting the evolutionary dynamics within *Cryobacterium*, and emphasizing the critical role of genomic adaptations for survival in glacial ecosystems. Gene flow analysis revealed that ecological units closely correlated with monophyletic clusters, with reduced genome identity and trait continuity between populations or ecological units, serve as a genetic barrier to horizontal gene transfer [32,43]. Insights from microbial population genomics can enhance community ecology, reinforcing the importance of deep genomic sequencing in assessing microbial diversity and revealing adaptation mechanisms in extreme environments.

## Data Availability

The genome sequences were deposited previously at DDBJ/ENA/GenBank and NMDC under the project numbers PRJNA421791 and NMDC10018147.

## Supplementary Materials

The following supporting information can be downloaded at: https://www.mdpi.com/article/10.3390/microorganisms14020326/s1, File S1: Manual for using PopCOGenT and custom-made scripts are available in our repository at https://github.com/environmental-genomes/Cryobacterium, accessed on 15 October 2023; Table S1: Cryobacterium genomes used in this study; Table S2: length_bias each population of Cryobacterium populations in this study; Table S3: Core gene sweep of Cryobacterium genomes in this study; Table S4: min AAI and min ANI in each population of Cryobacterium populations in this study.

## References

[1] Arevalo P., VanInsberghe D., Elsherbini J., Gore J., Polz M.F. (2019). A reverse ecology approach based on a biological definition of microbial populations. Cell.

[2] Gevers D., Cohan F.M., Lawrence J.G., Spratt B.G., Coenye T., Feil E.J., Stackebrandt E., Van de Peer Y., Vandamme P., Thompson F.L. (2005). Opinion: Re-evaluating prokaryotic species. Nat. Rev. Microbiol..

[3] Keeling P.J. (2024). Horizontal gene transfer in eukaryotes: Aligning theory with data. Nat. Rev. Genet..

[4] Hay M.C., Mitchell A.C., Soares A.R., Debbonaire A.R., Mogrovejo D.C., Els N., Edwards A. (2023). Metagenome-assembled genomes from High Arctic glaciers highlight the vulnerability of glacier-associated microbiota and their activities to habitat loss. Microb. Genom..

[5] Zhu S., Hong J., Wang T. (2024). Horizontal gene transfer is predicted to overcome the diversity limit of competing microbial species. Nat. Commun..

[6] Hunt D.E., David L.A., Gevers D., Preheim S.P., Alm E.J., Polz M.F. (2008). Resource partitioning and sympatric differentiation among closely related bacterioplankton. Science.

[7] Cadillo-Quiroz H., Didelot X., Held N.L., Herrera A., Darling A., Reno M.L., Krause D.J., Whitaker R.J. (2012). Patterns of gene flow define species of thermophilic Archaea. PLoS Biol..

[8] Kashtan N., Roggensack S.E., Rodrigue S., Thompson J.W., Biller S.J., Coe A., Ding H., Marttinen P., Malmstrom R.R., Stocker R. (2014). Single-cell genomics reveals hundreds of coexisting subpopulations in wild *Prochlorococcus*. Science.

[9] Stolyar S., Marx C.J. (2019). Align to Define: Ecologically Meaningful Populations from Genomes. Cell.

[10] Bohonak A.J. (1999). Dispersal, gene flow, and population structure. Q. Rev. Biol..

[11] Raymond-Bouchard I., Goordial J., Zolotarov Y., Ronholm J., Stromvik M., Bakermans C., Whyte L.G. (2018). Conserved genomic and amino acid traits of cold adaptation in subzero-growing Arctic permafrost bacteria. FEMS Microbiol. Ecol..

[12] Bradley J.A., Trivedi C.B., Winkel M., Mourot R., Lutz S., Larose C., Keuschnig C., Doting E., Halbach L., Zervas A. (2023). Active and dormant microorganisms on glacier surfaces. Geobiology.

[13] Kong W., Liu J., Ji M., Yue L., Kang S., Morgan-Kiss R.M. (2019). Autotrophic microbial community succession from glacier terminus to downstream waters on the Tibetan Plateau. FEMS Microbiol. Ecol..

[14] Tierney J.E., Zhu J., Li M., Ridgwell A., Hakim G.J., Poulsen C.J., Whiteford R.D.M., Rae J.W.B., Kump L.R. (2022). Spatial patterns of climate change across the Paleocene-Eocene Thermal Maximum. Proc. Natl. Acad. Sci. USA.

[15] Elser J.J., Wu C., González A.L., Shain D.H., Smith H.J., Sommaruga R., Williamson C.E., Brahney J., Hotaling S., Vanderwall J. (2020). Key rules of life and the fading cryosphere: Impacts in alpine lakes and streams. Glob. Change Biol..

[16] Marta S., Zimmer A., Caccianiga M., Gobbi M., Ambrosini R., Azzoni R.S., Gili F., Pittino F., Thuiller W., Provenzale A. (2023). Heterogeneous changes of soil microclimate in high mountains and glacier forelands. Nat. Commun..

[17] Anesio A.M., Lutz S., Chrismas N.A.M., Benning L.G. (2017). The microbiome of glaciers and ice sheets. npj Biofilms Microb..

[18] Bourquin M., Busi S.B., Fodelianakis S., Peter H., Washburne A., Kohler T.J., Ezzat L., Michoud G., Wilmes P., Battin T.J. (2022). The microbiome of cryospheric ecosystems. Nat. Commun..

[19] Ramón A., Esteves A., Villadóniga C., Chalar C., Castro-Sowinski S. (2023). A general overview of the multifactorial adaptation to cold: Biochemical mechanisms and strategies. Braz. J. Microbiol..

[20] Liu Q., Yang L.L., Xin Y.H. (2023). Diversity of the genus *Cryobacterium* and proposal of 19 novel species isolated from glaciers. Front. Microbiol..

[21] Liu Y., Shen L., Zeng Y., Xing T., Xu B., Wang N. (2020). Genomic Insights of *Cryobacterium* Isolated From Ice Core Reveal Genome Dynamics for Adaptation in Glacier. Front. Microbiol..

[22] Chaumeil P.A., Mussig A.J., Hugenholtz P., Parks D.H. (2022). GTDB-Tk v2: Memory friendly classification with the genome taxonomy database. Bioinformatics.

[23] Parks D.H., Imelfort M., Skennerton C.T., Hugenholtz P., Tyson G.W. (2015). CheckM: Assessing the quality of microbial genomes recovered from isolates, single cells, and metagenomes. Genome Res..

[24] Parks D.H., Rinke C., Chuvochina M., Chaumeil P.-A., Woodcroft B.J., Evans P.N., Hugenholtz P., Tyson G.W. (2017). Recovery of nearly 8000 metagenome-assembled genomes substantially expands the tree of life. Nat. Microbiol..

[25] Shen L., Liu Y., Allen M.A., Xu B., Wang N., Williams T.J., Wang F., Zhou Y., Liu Q., Cavicchioli R. (2021). Linking genomic and physiological characteristics of psychrophilic *Arthrobacter* to metagenomic data to explain global environmental distribution. Microbiome.

[26] Yang Z. (2006). Computational Molecular Evolution.

[27] Parks D.H., Chuvochina M., Rinke C., Mussig A.J., Chaumeil P.A., Hugenholtz P. (2022). GTDB: An ongoing census of bacterial and archaeal diversity through a phylogenetically consistent, rank normalized and complete genome-based taxonomy. Nucleic Acids Res..

[28] Asnicar F., Thomas A.M., Beghini F., Mengoni C., Manara S., Manghi P., Zhu Q., Bolzan M., Cumbo F., May U. (2020). Precise phylogenetic analysis of microbial isolates and genomes from metagenomes using PhyloPhlAn 3.0. Nat. Commun..

[29] Rosvall M., Bergstrom C.T. (2008). Maps of random walks on complex networks reveal community structure. Proc. Natl. Acad. Sci. USA.

[30] Bozdag G.O., Ono J. (2022). Evolution and molecular bases of reproductive isolation. Microb. Biotechnol..

[31] Kryazhimskiy S., Plotkin J.B. (2008). The population genetics of dN/dS. PLoS Genet..

[32] VanInsberghe D., Arevalo P., Chien D., Polz M.F. (2020). How can microbial population genomics inform community ecology?. Philos. Trans. R. Soc. B.

[33] Liu Q., Song W.Z., Zhou Y.G., Dong X.Z., Xin Y.H. (2020). Phenotypic divergence of thermotolerance: Molecular basis and cold adaptive evolution related to intrinsic DNA flexibility of glacier-inhabiting *Cryobacterium* strains. Environ. Microbiol..

[34] Yang Z., Bielawski J.P. (2000). Statistical methods for detecting molecular adaptation. Trends. Ecol. Evol..

[35] Sasgen I., Steinhoefel G., Kasprzyk C., Matthes H., Westermann S., Boike J., Grosse G. (2024). Atmosphere circulation patterns synchronize pan-Arctic glacier melt and permafrost thaw. Commun. Earth Environ..

[36] Sundaram S., Karakaya H., Scanlan D.J., Mann N.H. (1998). Multiple oligomeric forms of glucose-6-phosphate dehydrogenase in cyanobacteria and the role of OpcA in the assembly process. Microbiology.

[37] Duong F., Wickner W. (1997). Distinct catalytic roles of the SecYE, SecG and SecDFyajC subunits of preprotein translocase holoenzyme. EMBO J..

[38] Bortoluzzi A., Muskett F.W., Waters L.C., Addis P.W., Rieck B., Munder T., Schleier S., Forti F., Ghisotti D., Carr M.D. (2013). *Mycobacterium tuberculosis* RNA polymerase-binding protein A (RbpA) and its interactions with sigma factors. J. Biol. Chem..

[39] Vanoni M.A., Verzotti E., Zanetti G., Curti B. (1996). Properties of the recombinant beta subunit of glutamate synthase. Eur. J. Biochem..

[40] van den Heuvel R.H., Curti B., Vanoni M.A., Mattevi A. (2004). Glutamate synthase: A fascinating pathway from L-glutamine to L-glutamate. Cell Mol. Life Sci..

[41] Bush M.J., Bibb M.J., Chandra G., Findlay K.C., Buttner M.J. (2013). Genes Required for Aerial Growth, Cell Division, and Chromosome Segregation Are Targets of WhiA before Sporulation in Streptomyces venezuelae. Microb. Biotechnol..

[42] Chen X., Court D.L., Ji X. (1999). Crystal structure of ERA: A GTPase-dependent cell cycle regulator containing an RNA binding motif. Proc. Natl. Acad. Sci. USA.

[43] Shen L., Liu Y., Chen L., Lei T., Ren P., Ji M., Song W., Lin H., Su W., Wang S. (2024). Genomic basis of environmental adaptation in the widespread poly-extremophilic Exiguobacterium group. ISME J..

