# Supplementary material for: Population Ecology of Glacier Bacteria from the View of Gene Flow in Cryobacterium
Source: Microorganisms. 2026 Jan 30;14(2):326. doi: 10.3390/microorganisms14020326 (PMC12943682; doi:10.3390/microorganisms14020326)
Supplement: Supplementary file 1 [file microorganisms-14-00326-s001.zip › Additional Methods.pdf]

Integrated Manual: PopCOGenT Setup and Core Gene Sweep Analysis

Many scholars have made evaluations of PopCOGenT:

PopCOGenT is unique in its ability to detect gene transfer between genomes. It can detect the transfer of all genomic regions shared by any two strains, including any extrachromosomal mobile genetic material.

Conceptual Background: Reverse Ecology and PopCOGenT

1. Traditional Ecology vs. Reverse Ecology

- **Traditional Ecology**:

  Top-down approach starting with ecologically distinct populations (e.g., temperature tolerance, substrate utilization) → Identifies genetic drivers of functional divergence.

- **Reverse Ecology**:

  Bottom-up approach starting with genomic data → Infers ecological/metabolic traits directly from genes → Reveals hidden functional boundaries between populations/species.

2. Gene Flow in Population Genetics

- **Definition**: Transfer of genetic material between populations (*horizontal gene transfer, HGT*) altering population genetic structure.
- **Key Insight**:
  - High gene flow **within** populations → Shared adaptations.
  - Low gene flow **between** populations → Functional divergence.

3. PopCOGenT: Reverse Ecology in Action

PopCOGenT bridges genomics and ecology through **HGT-driven network analysis**:

Step	Mechanism	Biological Significance
**1. Detect Recent HGT**	Quantifies length/frequency of shared HGT events	Measures *genetic connectivity*
**2. Build Gene Flow Network**	Nodes = Genomes; Edges = HGT strength	Maps *population structure*
**3. Cluster Identification**	Splits network into isolated communities (e.g., Infomap)	Defines **populations** (genetically isolated units)
**4. Ecological Inference**	Functional traits propagate via HGT within clusters	Populations ≈ **Ecologically cohesive units**

Why This is Reverse Ecology?

- **Input**: Raw genomes (no prior ecological data).
- **Output**:
  - Populations defined by *gene flow barriers* (HGT discontinuity).
  - Inferred ecological roles (via HGT-transferred functions like nutrient uptake, stress resistance).
- **Key Implication**:

> Genomic boundaries (HGT networks) ≡ Functional boundaries (ecological niches).

Example Workflow:

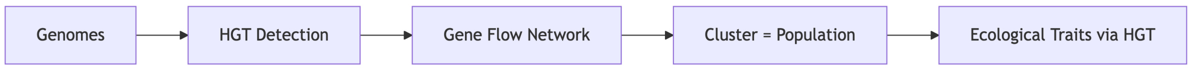

PopCOGenT transforms genomes into ecologically meaningful units without phenotypic pre-screening.

Configuration

I. PopCOGenT Installation & Setup

1.1 System Preparation

```
# Create conda environment
conda config --set restore_free_channel true
conda env create -f PopCOGenT.yml

# Compile Infomap
cd PopCOGenT-master/Infomap
make
```

1.2 Configuration

Edit files:

1. `mugsyenv.sh`:

   ```
   export MUGSY_INSTALL=/absolute/path/to/mugsy
   ```

2. `src/PopCOGenT/config.sh`:

   ```
   genome_dir="/path/to/genomes/"  # Must end with /
   base_name="output_prefix"
   # Paths to tools (set once)
   mugsy_path="..."
   mugsy_env="..."
   infomap_path="..."
   ```

1.3 Path Specifications

Location	Path
Server	`~/Jihua/PopCOGenT-master`
Local Lab Machine	`~/Jihua/PopCOGenT-master`

> Note: Update paths if folder names change.

II. Running PopCOGenT

2.1 Execution Workflow

```
cd /path/to/PopCOGenT-master/src/PopCOGenT/
conda activate PopCOGenT
./PopCOGenT.sh  # Outputs in ./output/
```

2.2 Output Files

File Type	Location	Use Case
`.net`	`output/infomap_out/`	Network visualization
`.maf`	`output/alignments/`	Genome alignments

III. Downstream Analysis

3.1 Network Visualization

1. Access Infomap Online
2. Load `.net` file → "Run Infomap" → "Show network"

3.2 Phylogenetic Tree (PhyloPhlAn)

```
phylophlan \\
  --input genome_dir/align \\
  -d phylophlan \\
  -t a \\
  --diversity low \\
  -f ~/supermatrix_aa.cfg \\
  --nproc 32 \\
  --min_num_markers 100 \\
  -o /output/directory
```

Visualization: Open `.tre` in FigTree → Color by clusters

3.3 Geographic Mapping

Use `sampleMap.html` with:

- Latitude/longitude table
- Cluster assignments from PopCOGenT

IV. Core Gene Sweep Analysis

> Lab machine only

4.1 Toolchain Overview

Tool	Function	Critical Parameters
**MUGSY**	Multi-genome alignment (MAF format)	`--max-mem 16G` (large genomes)
**PhyML**	Maximum-likelihood trees	`-m HKY85` (simpler model)
**APE (R)**	Tree distances & monophyly tests	`is.monophyletic()`
**Custom Python**	SNP calling, diversity (π), window analysis	`gap_prop_thresh=0.2`

4.2 Sweep Detection Workflow

Step 1: Data Preparation

```
# Standardize FASTA headers
sed -i 's/[[:punct:]]//g' *.fasta

# Align genomes
mugsy genome*.fa --directory ALIGN_OUT --prefix core_align

# Filter alignments (Python)
filter_alignments.py --gap_thresh 0.2 --min_len 500
```

Step 2: Core Region Scanning

```
# Per-LCB metrics
conservation = calc_invariant_sites(LCB)
snp_count = poly_count(LCB, min_maf=0.05)
pi_value = calc_pop_div(LCB, window_size=1000)
```

Step 3: Sweep Detection

```
# In R (using APE)
focus_tips <- c("StrainA", "StrainB")
is.monophyletic(tree, tips = focus_tips)  # Monophyly test
branch_score <- focus_branch / tree_length  # Branch length ratio
```

```
# π reduction (Python)
background_pi = genome_wide_pi_distribution()
low_ci = stats.binom.interval(0.95, background_pi)
sweep_windows = [win for win in windows if win.pi < low_ci[0]]
```

Step 4: Result Consolidation

```
merged_regions = concatenate_windows(sweep_windows, max_gap=5000)
final_hits = filter(merged_regions,
                    branch_score > 0.8,
                    monophyly == True,
                    p_value < 0.05)
```

4.3 Script Execution Order

1. `phybreak1`: Project setup
2. `phybreak2`: Data preprocessing
3. `phybreak3`: Phylogeny building (PhyML)
4. `phybreak4`: Tree analysis (run `phybreak4.R` manually)
5. `phybreak5-7`: Diversity calculations & sweep calling

> Critical Checks:
>
> - Reference genome must have **exactly 1 contig**
> - Validate file paths in all scripts

V. Output Interpretation

Core Sweep Results (BED Format)

Column	Description	Threshold
`BranchLengthScore`	Focus branch / total tree length	>0.8
`Meanπ`	Nucleotide diversity in window	<5% CI
`MonophylyFlag`	1=monophyletic, 0=non-monophyletic	1
`P-value`	Binomial test vs background π	<0.05

VI. Troubleshooting Guide

**Issue**	**Solution**
**MUGSY crashes**	1. Sanitize FASTA headers<br>2. Increase `--max-mem`
**PhyML fails convergence**	1. Simplify model (`-m HKY85`)<br>2. Reduce bootstraps (`-n 50`)
**Low SNP sensitivity**	Adjust minor allele frequency in `poly_count()`
`phybreak4.R` errors	Run outside PopCOGenT conda environment
**Missing output files**	Check path in `config.sh` and disk space

> Reproducibility Tip: Document all parameters in analysis_log.txt:

```
# Example log entry
MUGSY: v1.2.3, --max-mem 32G
PhyML: v3.3, -m GTR -n 100
Sweep params: gap_thresh=0.2, min_len=500, window_size=1000
```
